# Herencia de las enfermedades comunes

**Published:** 2022-05-01

**Authors:** Jaime Bernal Villegas

**Affiliations:** 1 Universidad del Sinú, sede Cartagena de Indias, Colombia Universidad del Sinú Universidad del Sinú Cartagena de Indias Colombia

Corría el año de 1962 y el navío norteamericano *USS Little Rock* se encontraba en un crucero por el Lejano Oriente. En el último puerto, seis meses antes de llegar de nuevo a casa, la tripulación decidió hacer un pícnic, pero dada la frecuencia de enfermedades infecciosas en esa región, el personal de cocina tomó todas las precauciones posibles. Poco después del pícnic, el crucero levó anclas y, en las siguientes horas, aparecieron los primeros casos de enfermedad diarreica. De los 1.276 tripulantes, 602 sufrieron la enfermedad, transmitida por dos de los cocineros que ocultaron su padecimiento para no perder la bajada a tierra firme. En las siguientes dos semanas se observó que diez de los enfermos desarrollaron la enfermedad de Reiter. La conclusión obvia fue que el agente patógeno involucrado, la bacteria *Shigella* spp., desencadenaba en alguna forma la artritis reactiva de Reiter.

Esta historia fue debidamente publicada ^1^, pero también olvidada durante 13 años, hasta que se conoció la asociación entre el antígeno HLA-B27 y la enfermedad de Reiter, Andrei Calin y James Fries ^2^. Entonces, comenzó la tarea de buscar a aquellos tripulantes que la habían contraído; encontraron a cinco, de los cuales cuatro resultaron positivos para el HLA B27, en tanto que uno de ellos, que había tenido la enfermedad, dio negativo y se encontraba libre de todo síntoma. El caso del *USS Little Rock* permite sacar varias conclusiones, la más importante y obvia es que tanto el B27 como la diarrea bacteriana son elementos instrumentales en la aparición del síndrome de Reiter. Ahora bien, dado que no todos los individuos con diarrea y positivos para el B27 tuvieron el síndrome, en tanto que uno de los negativos sí lo presentó, estaba claro que debía haber otros factores involucrados.

Pocos años después documentamos la asociación de la diabetes de tipo 1 con el factor B del complemento sérico, cuyo gen se encuentra en el cromosoma 6, cercano a los genes del HLA ^3^. Más recientemente, describimos también la asociación del HLA con el prurigo actínico, una fotodermatosis inducida por la exposición a la luz solar, más común en indios americanos y particularmente frecuente entre los indígenas chimila, cerca de Fundación, en el departamento del Magdalena ^4^^,^^5^. Estos son ejemplos de enfermedades comunes cuya etiología tiene un componente genético y otro ambiental, lo que en, términos de la genética clásica, se conoce como herencia multifactorial o poligénica. En este tipo de herencia se implican múltiples loci que contribuyen al fenotipo, cada uno de ellos con un efecto muy pequeño, pero cuya suma determinaría el riesgo individual y permitiría detectar los individuos con gran riesgo de desarrollar la enfermedad.

La secuenciación del genoma humano ha hecho posible comprobar que en él existen muchísimas variaciones entre individuos, las cuales, al contrario de las mutaciones, son muy frecuentes y de repercusión funcional posiblemente muy limitada o nula; de hecho, la mayor parte de esas variaciones, o polimorfismos, se encuentra en regiones no codificantes del genoma. Las más conocidas son: los polimorfismos de nucleótido único (SNP), que consisten en el cambio de una base por otra y se dan por millones en el genoma; los polimorfismos de repetición en tándem, en los que una secuencia pequeña de nucleótidos (STR) o de número variable (VNTR) se repite en el genoma, y los polimorfismos de variación en el número de copias (CNV), que son segmentos de variaciones interindividuales en el número de copias de un determinado gen.

El análisis de estos polimorfismos y su posible asociación con enfermedades, comenzó por identificar genes candidatos y determinar si sus polimorfismos se asociaban con la presencia de la enfermedad. Sin embargo, la fuerza de las asociaciones de genotipo y fenotipo ha sido débil y su observación difícilmente se repite. El desarrollo de los microarreglos ha facilitado el análisis simultáneo, rápido y a bajo costo, de miles de SNP. Además, esta aproximación permite establecer las asociaciones sin tener que buscar los genes candidatos, pues los SNP están distribuidos en todos los cromosomas, por lo que se analiza, entonces, todo el genoma.

Estos microarreglos permiten analizar rápidamente todo el genoma de un grupo de pacientes con determinada enfermedad y compararlo con otro grupo similar sin la enfermedad. Tales estudios se llaman de asociación genómica, o *Genome-Wide Association Studies* (GWGA). Aunque se ha generado una gran expectativa sobre sus resultados, y son miles las series de pacientes y personas estudiadas (ver la lista en www.genome.gov/GWAStudies), hay posiciones encontradas sobre los hallazgos. Por un lado, los estimativos de riesgo o vulnerabilidad varían, por lo general, a medida que se se van caracterizando mejor las manifestaciones clínicas de la enfermedad.

Esto se hace evidente en el caso de la diabetes, cuyas primeras estimaciones de heredabilidad se calcularon en 0,75 ^6^, luego en 0,88 para la diabetes de tipo 1 ^7^ y en 0,26 para la de tipo 2 ^8^; en tanto que la diabetes de tipo MODY (*Maturity Onset Diabetes of the Young*), que es de herencia dominante autosómica, tiene cerca de 14 subtipos ^9^. Debe recordarse, también, que se desconocen los factores ambientales y su papel en el desarrollo de la mayoría de las enfermedades, los cuales, además, pueden variar enormemente entre las distintas poblaciones y generaciones, por lo que es imposible tenerlos en cuenta para el cálculo de los riesgos. Pareciera, entonces, que el modelo de estudio del componente genético en la etiología de las enfermedades multifactoriales se está agotando y ya se requieren nuevas estrategias para continuar avanzando.

Por otro lado, en los últimos 20 años ha aparecido un nuevo actor en el panorama de la relación entre el genotipo y el fenotipo: la epigenética, definida como el estudio de los cambios heredables en la función de los genes que no se pueden explicar por cambios en la estructura del ADN. Los mecanismos epigenéticos ordenan la expresión o el silenciamiento de genes dentro de la célula, lo cual implica que cumplen una función en la embriogénesis y en los procesos de diferenciación celular, entre otros ^10^.

No es objeto de esta nota entrar en la descripción molecular de los mecanismos de la epigenética, baste decir que estos (metilación del ADN ^11^, modificaciones postranslacionales de las proteínas de la histona y el ARN no codificador) responden directamente a cambios en el ambiente, lo cual quiere decir que no solo lideran los procesos de expresión génica, sino que también responden al ambiente que rodea al individuo.

Por mucho tiempo se pensó que el ambiente que incidía en la etiología de las enfermedades complejas era externo al individuo. Hoy se sabe que empieza en el interior de la célula y que la epigenómica del individuo cambia con la exposición a agentes externos, como la dieta, el ejercicio y los hábitos cotidianos, por ejemplo, fumar o beber alcohol. Ha sido en el campo del diagnóstico y el tratamiento del cáncer donde se ha visto más claramente el posible impacto de la epigenómica, y ya se tienen algunos mapas de regiones del genoma que son hipermetiladas o hipometiladas en el transcurso del desarrollo de un cáncer ^12^. Parece claro, entonces, que, a pesar de las luces que se perciben, la tarea de dilucidar de mejor manera la extraordinaria complejidad de la enfermedad humana se ha hecho más compleja.

## References

[1] Noer HR. (1966). An “experimental” epidemic of Reiter syndrome. JAMA.

[2] Calin A, Fries J. (1976). An “experimental” epidemic of Reiter syndrome revisited: Follow-up evidence on genetic and environmental factors. Ann Intern Med.

[3] Bernal JE, Ellis DA, Haigh J. (1979). Bf in insulin-dependant diabetes. Lancet.

[4] Bernal JE, Durán-de Rueda MM, Ordóñez CP, Durán C, de Brigard D. (1990). Actinic prurigo among the Chimila indians in Colombia. HLA studies. J Am Acad Dermatol.

[5] Durán MM, Bernal J. (1992). HLA typing in actinic prurigo. J Am Acad Dermatol.

[6] Harvald B, Hauge M. (1965). Hereditary factors elucidated by twin studies. En: Neel J, Shaw M, Schull W, *et al*. (Editors) Genetics to Epidemiological Studies of Chronic Diseases.

[7] Hyttinen V, Kaprio J, Kinnunen L, Koskenvuo M, Tuomilehto J. (2003). Genetic liability of type 1 diabetes and the onset age among 22,650 Young Finnish twin pairs: A nationwide follow-up study. Diabetes.

[8] Poulsen P, Kyvik KO, Vaag A, Beck-Nielsen H. (1999). Heritability of type II (non-insulin-dependent) diabetes mellitus and abnormal glucose tolerance - a population-based twin study. Diabetologia.

[9] Urakami T. (2019). Maturity-onset diabetes of the young (MODY): Current perspectives on diagnosis and treatment. Diabetes Metab Syndr Obes.

[10] Heijmans BT, Tobi EW, Stein AD, Putter H, Blauw GJ, Susser ES (2008). Persistent epigenetic differences associated with prenatal exposure to famine in humans. Proc Natl Acad Sci U S A.

[11] Tobi EW, Lumey LH, Talens RP, Kremer D, Putter H, Stein AD (2009). DNA methylation differences after exposure to prenatal famine are common and timing- and sex-specific. Hum Mol Genet.

[12] Fraga MF, Herranz M, Espada J, Ballestar E, Paz MF, Ropero S (2004). A mouse skin multistage carcinogenesis model reflects the aberrant DNA methylation patterns of human tumors. Cancer Res.

